# Early Mortality Prediction in Intensive Care Unit Patients Based on Serum Metabolomic Fingerprint

**DOI:** 10.3390/ijms252413609

**Published:** 2024-12-19

**Authors:** Rúben Araújo, Luís Ramalhete, Cristiana P. Von Rekowski, Tiago A. H. Fonseca, Luís Bento, Cecília R. C. Calado

**Affiliations:** 1 NMS—NOVA Medical School, FCM—Faculdade de Ciências Médicas, Universidade NOVA de Lisboa, Campo dos Mártires da Pátria 130, 1169-056 Lisbon, Portugal; rubenalexandredinisaraujo@gmail.com (R.A.);; 2 CHRC—Comprehensive Health Research Centre, Universidade NOVA de Lisboa, 1150-082 Lisbon, Portugal; 3 ISEL—Instituto Superior de Engenharia de Lisboa, Instituto Politécnico de Lisboa, Rua Conselheiro Emídio Navarro 1, 1959-007 Lisbon, Portugal; 4 IPST—Instituto Português do Sangue e da Transplantação, Alameda das Linhas de Torres—nr.117, 1769-001 Lisbon, Portugal; 5 iNOVA4Health—Advancing Precision Medicine, RG11, Reno-Vascular Diseases Group, NMS—NOVA Medical School, FCM—Faculdade de Ciências Médicas, Universidade NOVA de Lisboa, 1169-056 Lisbon, Portugal; 6 Intensive Care Department, ULSSJ—Unidade Local de Saúde São José, Rua José António Serrano, 1150-199 Lisbon, Portugal; 7 Integrated Pathophysiological Mechanisms, CHRC—Comprehensive Health Research Centre, NMS—NOVA Medical School, FCM—Faculdade de Ciências Médicas, Universidade NOVA de Lisboa, Campo Mártires da Pátria 130, 1169-056 Lisbon, Portugal; 8 iBB—Institute for Bioengineering and Biosciences, i4HB—The Associate Laboratory Institute for Health and Bioeconomy, IST—Instituto Superior Técnico, Universidade de Lisboa, Av. Rovisco Pais, 1049-001 Lisbon, Portugal

**Keywords:** ICU mortality prediction, serum biomarkers, FTIR spectroscopy, omics

## Abstract

Predicting mortality in intensive care units (ICUs) is essential for timely interventions and efficient resource use, especially during pandemics like COVID-19, where high mortality persisted even after the state of emergency ended. Current mortality prediction methods remain limited, especially for critically ill ICU patients, due to their dynamic metabolic changes and heterogeneous pathophysiological processes. This study evaluated how the serum metabolomic fingerprint, acquired through Fourier-Transform Infrared (FTIR) spectroscopy, could support mortality prediction models in COVID-19 ICU patients. A preliminary univariate analysis of serum FTIR spectra revealed significant spectral differences between 21 discharged and 23 deceased patients; however, the most significant spectral bands did not yield high-performing predictive models. By applying a Fast-Correlation-Based Filter (FCBF) for feature selection of the spectra, a set of spectral bands spanning a broader range of molecular functional groups was identified, which enabled Naïve Bayes models with AUCs of 0.79, 0.97, and 0.98 for the first 48 h of ICU admission, seven days prior, and the day of the outcome, respectively, which are, in turn, defined as either death or discharge from the ICU. These findings suggest FTIR spectroscopy as a rapid, economical, and minimally invasive diagnostic tool, but further validation is needed in larger, more diverse cohorts.

## 1. Introduction

The study of pandemics, such as Coronavirus Disease 2019 (COVID-19) caused by severe acute respiratory syndrome coronavirus 2 (SARS-CoV-2), remains crucial even as the world has entered a post-emergency phase [1]. Despite the global health community’s significant efforts to contain the virus and minimize fatalities, the long-term impacts of COVID-19 continue to challenge healthcare systems [2,3]. A PubMed search query using the keywords “COVID-19”, “SARS-CoV-2”, and related terms revealed that from 2020 to 2024 (accessed on 16 October 2024), there has been a decline in COVID-19-related research publications, with a 66.6% decrease in the number of articles published between the peak in 2021 (137,141) and 2024 (45,761). However, the persistence of the virus, combined with emerging variants, underscores the need for continued investigation, particularly when it comes to Intensive Care Unit (ICU) patient outcomes [4]. It is essential to maintain focus on this research to improve preparedness for future pandemics and to refine ongoing strategies for managing the still poorly understood long-term effects of COVID-19 [5,6]. These persistent changes, observable even months after hospitalization, are reshaping our understanding of long COVID, with some proposing that it warrants classification as a form of brain injury due to its neurological impacts [7,8].

At the peak of the COVID-19 pandemic, ICUs worldwide experienced immense pressure, often operating at or beyond capacity [9]. As such, the ability to predict mortality in ICU patients, particularly those with severe infections, would allow for timely interventions and better resource allocation. Early identification of at-risk patients could improve survival rates by guiding targeted treatment strategies, a necessity during times of critical care such as the COVID-19 pandemic. This is particularly important given that ICU patients are often overloaded with a wide range of complications, making it difficult to predict outcomes based on conventional clinical parameters [10]. Therefore, integrating molecular approaches to enhance predictive accuracy is a promising area of exploration. The need for reliable prediction models in ICUs has long been recognized, with existing clinical scoring systems—such as the APACHE III, SOFA, and SAPS II scores—being widely used to assess illness severity and predict mortality [11]. However, while these models perform well for benchmarking ICU performance and comparing outcomes between hospitals, their precision in predicting individual patient outcomes is limited due to their reliance on more conventional variables, wherein clinical data may not fully capture the evolving physiological status of the patient, with area under the receiver operating characteristic (ROC) curve (AUC) values typically ranging, in percentage, from 70% to 80% [12,13,14].

Several studies have explored the potential of using biofluids to identify biomarkers for predicting mortality. Traditional techniques have identified biomarkers associated with poor outcomes in critically ill patients. These include soluble urokinase-type plasminogen activator receptor (suPAR), which reflects immune activation, and inflammatory cytokine IL-6. However, these biomarkers often suffer from limitations due to the complexity and high variability in ICU populations, leading to inconsistent predictive accuracy and limited clinical applicability [15,16]. This has led to a growing interest in integrating omics approaches, which encompass a wide array of scientific fields, each aiming to provide a large-scale set of defined molecules [17]—such as genomics [18], transcriptomics [19], proteomics [20,21], and metabolomics [22]—to provide a more comprehensive understanding of biological systems.

Omics, when applied to biofluids like serum, present the advantage of using a biofluid obtained by minimally invasive procedures while capturing the organism’s pathophysiological state [23,24]. Among all omics, metabolomics offers a significant advantage as it represents the system’s phenotype, providing a holistic view of the organism’s metabolic state [25]. However, metabolomics, like other omics, is usually based on expensive, complex, laborious, and time-consuming methods, ultimately limiting studies to low-dimension populations and, consequently, leading to less robust predictive models [26]. An alternative, cost-effective method for rapidly acquiring the metabolic status of a system is through biofluid analysis using Fourier Transform Infrared (FTIR) spectroscopy.

FTIR spectroscopy is a powerful analytical tool capable of detecting subtle biochemical changes by analyzing the vibrational modes of molecules within biological samples. This technique is particularly effective in the mid-infrared region (400 to 4000 cm^−1^), where it captures the fundamental vibrational signatures of a wide range of functional groups present in complex biological samples. FTIR spectroscopy has been applied across various biomedical fields, allowing for the analysis of diverse human biofluids such as blood, saliva, urine, tears, and sweat for medical diagnostics [27,28,29,30]. It has been applied to the diagnosis of conditions such as cancers [31], rheumatoid arthritis [32], and neurological [33,34,35] and immune system disorders [36]. In clinical contexts, FTIR spectroscopy’s high sensitivity and specificity in capturing the metabolic state of a patient make it highly applicable in ICU settings, where accurate prediction of mortality is essential [37]. By analyzing the molecular signature of biofluids such as serum, FTIR spectroscopy provides a non-invasive method for evaluating systemic metabolic changes associated with severe infections like COVID-19.

In the search for biomarkers, serum is typically preferred over plasma due to its stability and ease of storage, particularly at low temperatures. Serum lacks clotting factors present in plasma, which can introduce variability in molecular analyses. This makes serum a more reliable biofluid for tracking the physiological state of a patient over time, which is crucial for capturing dynamic changes in an ICU patient, allowing for more robust measurements, particularly in FTIR-spectroscopy-based studies, where small biochemical changes can provide critical insights into disease progression [38].

The primary aim of this preliminary study was to develop predictive models of ICU mortality, using FTIR spectroscopy to analyze the serum metabolic profiles of 44 ICU COVID-19 patients. Of these, 23 patients were deceased, while the remaining were discharged from the ICU. By combining FTIR spectroscopy with advanced machine learning algorithms, this study seeks to improve the accuracy of mortality prediction and provide timely insights that could enhance clinical decision-making.

## 2. Results and Discussion

### 2.1. Current Mortality Risk Associated with COVID-19

**Figure 1:**
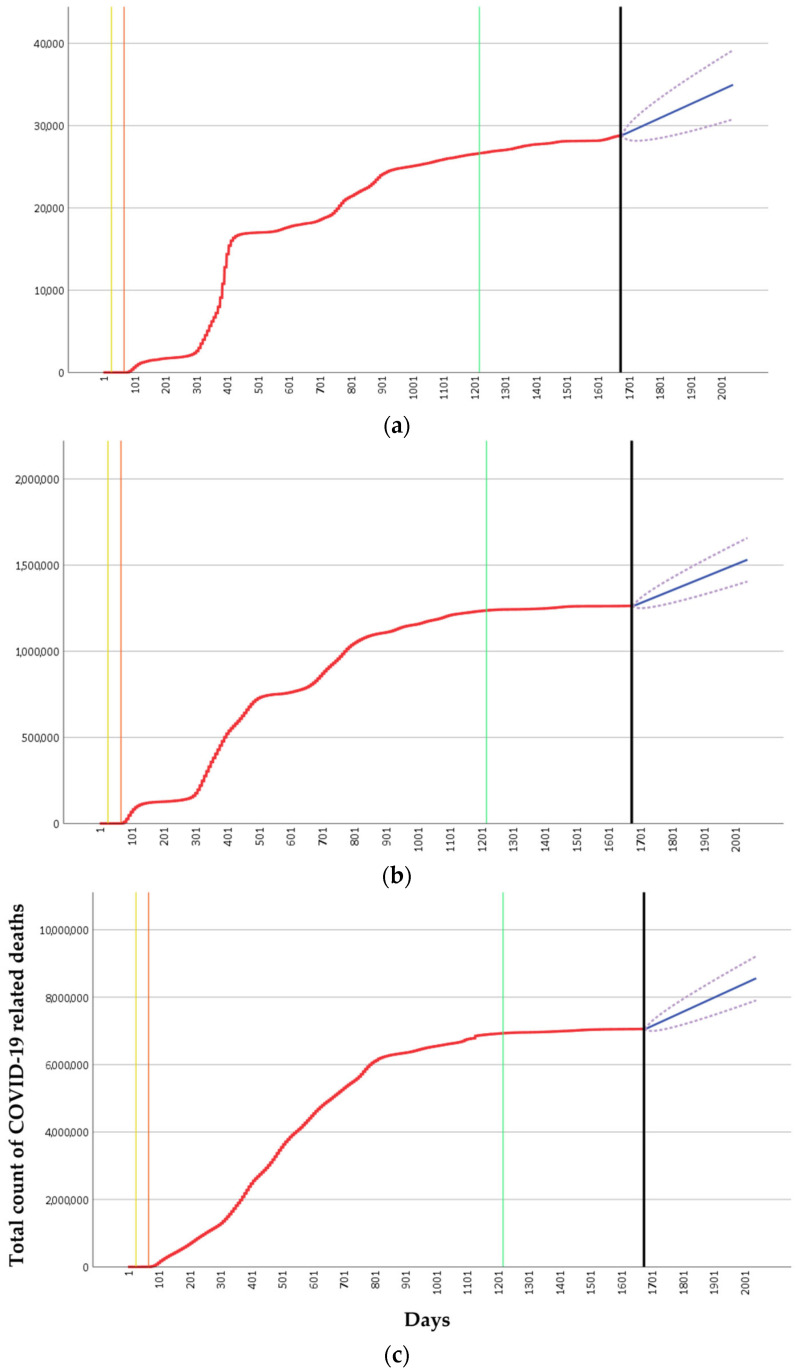
Total count of daily deaths (red lines) due to COVID-19 in (**a**) Portugal, (**b**) the EU, and (**c**) worldwide from 5 January 2020 to 4 August 2024 (black vertical line). The remaining vertical lines represent the following: yellow—WHO declares COVID-19 a Public Health Emergency of International Concern (30 January 2020); orange—WHO declares COVID-19 a pandemic (11 March 2020); green—WHO declares the end of the emergency phase (5 May 2023). The blue lines represent the predicted total count of COVID-19-related deaths based on an ARIMA forecast for the next 365 days after the last of the observed days (4 August 2024). The purple dotted lines represent both the Lower Confidence Level and Upper Confidence Level.

To evaluate the relevance of current and future mortality risk by COVID-19, the number of deaths due to COVID-19 was forecasted from 4 August 2024 to the following year (4 August 2025), using AutoRegressive Integrated Moving Average (ARIMA) models for Portugal, the 27 countries that make up the European Union (EU), and the global population (Figure 1). The current analysis was based on weekly data from the World Health Organization (WHO), compiled through the Our World in Data (OWID) GitHub repository [39,40]. It is worth noting that Johns Hopkins University (on which WHO based their data) ceased daily COVID-19 updates on 10 March 2023 [41], leading WHO to transition to weekly updates from 25 August 2023, while OWID updates extend up to 4 August 2024. This made OWID the choice for this analysis as it contained the most up-to-date datasets regarding daily ICU deaths across all countries. Information about cross-country COVID-19 testing and a global database of COVID-19 vaccinations can also be consulted [42,43]

On 30 January 2020, when the WHO declared COVID-19 to be a Public Health Emergency of International Concern (PHEIC), in Portugal, reported deaths remained at zero, with 6 reported in the EU and 62 globally. By 5 May 2023, when the COVID-19 emergency phase ended, Portugal’s confirmed deaths reached 26,604, with the EU and global death counts reported as 1,235,954 and 6,927,839, respectively. From the end of the pandemic, 4 August 2024 (black line in Figure 1), Portugal showed an 8% growth in COVID-19-related deaths, while the EU and global levels exhibited growth rates of approximately 2%. These increases highlight the ongoing impact of the virus. It may also reflect the persistent vulnerabilities among certain populations and residual impacts of the pandemic, a grim reminder that COVID-19 remains a public health concern even post-pandemic.

Using historical daily death count data observed up to 4 August 2024, total deaths were forecast one year into the future, up to 4 August 2025, using an ARIMA model. Portugal’s forecast cumulative deaths for this period were estimated to reach 34,958, reflecting a 21% increase from the previous year (4 August 2024), with a lower confidence level (LCL) of 30,757 (an increase of 7%) and an upper confidence level (UCL) of 39,160 (a 36% increase). In the EU, predicted deaths are 1,531,063, which represents a 21% increase, with an LCL of 1,404,590 (11% increase) and a UCL of 1,657,535 (31% increase). Globally, the model estimates 8,559,570 deaths, an increase of 21%, comparatively to the last observed date, with an LCL of 7,905,330 (12% increase) and a UCL of 9,213,811 (31% increase).

The slightly higher projected increase in deaths in Portugal for 2025, based on both the forecasted and UCL values, when compared to both the EU and global levels, may reflect regional differences in testing, reporting, and public health practices [44,45].

In Portugal, for example, ongoing testing among vulnerable populations, such as the elderly, may contribute to more accurate reporting. Combined with Portugal’s lower post-pandemic vaccination rates and a public perception that COVID-19 is no longer a threat, these factors likely contribute to the documented increase in COVID-19-related deaths, especially after the WHO declared the end of the emergency phase.

The continued rise in deaths suggests that COVID-19 remains an ongoing concern. Accurate forecasting models could then inform strategic healthcare planning and resource allocation, particularly in ICU settings where anticipating resource needs is crucial [46]. Therefore, it remains essential for the current COVID-19 situation and future pandemics to develop tools for monitoring and managing these types of patients, especially those requiring critical human and material resources, as seen in ICUs. In this study, predictive models of mortality were developed for COVID-19 patients in an ICU at a central hospital in Lisbon, Portugal.

### 2.2. Study Population Characteristics

This study included 44 ICU patients from a central hospital in Lisbon, of whom 23 were deceased, and the remaining were discharged from the ICU. The two groups of patients (discharged from the ICU, i.e., survivors and deceased patients) did not differ statistically (*p* > 0.01) in relation to demographic characteristics (age, gender) and clinical variables such as Extracorporeal Membrane Oxygenation (ECMO) and relevant comorbidities [47,48] such as arterial hypertension, diabetes mellitus, and dyslipidemia (Table 1). This allows the effect of confounding variables to be minimized. All patients were on invasive mechanical ventilation (IMV), according to the observations for critically ill COVID-19 patients. Blood samples were collected from 12 November 2020 to 24 September 2021 as part of the ICU Standard of Care (SOC). Initially, samples were collected daily until 3 May 2021, when, due to the high workload on ICU staff during the COVID-19 pandemic, daily sampling was discontinued. From this date forward, samples were collected every two days, and weekends were excluded to alleviate staff burden. The final dataset was shaped by this adjustment as only patients with complete samples from all three selected timeframes (the first 48 h of ICU admission, seven days prior to the outcome, and the day of the outcome) were included in the study. For further details regarding the study population and sample processing, please refer to Section 3.1 and Section 3.2.

### 2.3. Univariate Spectral Analysis

**Figure 2:**
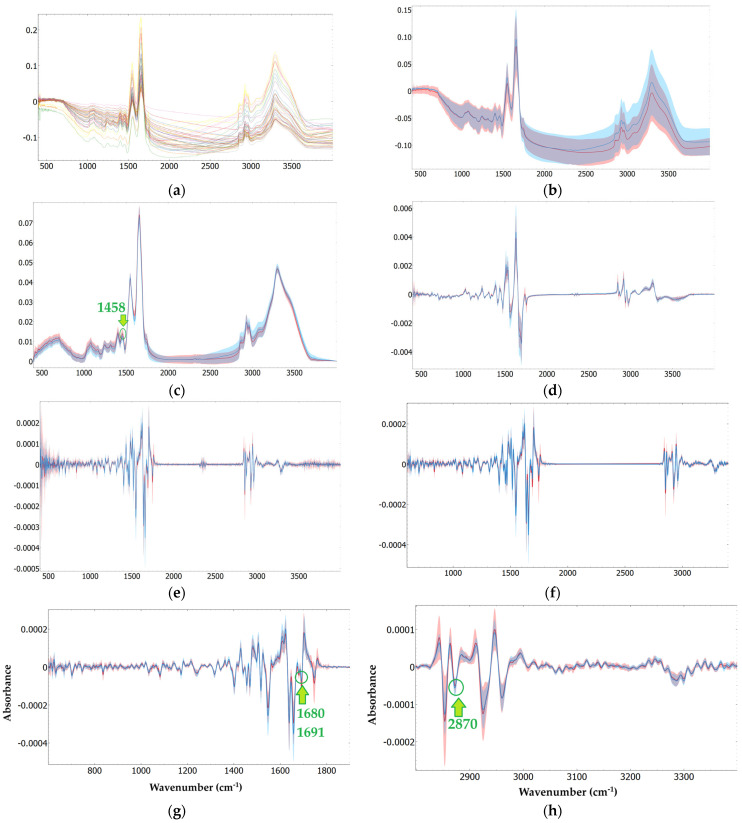
Serum spectra of discharged (blue line) and deceased (red line) patients on the day of outcome: (**a**) All individual spectra for each group (23 deceased and 21 discharged); (**b**) The average spectra for each group (colored shadows represent the range of all individual spectra for each group). The following spectra preprocessing steps were applied: (**a**,**b**): None; (**c**) Baseline correction and unit vector normalization; (**d**) First derivative; (**e**) Second derivative; (**f**) Second derivative without low signal-to-noise regions; (**g**) Second derivative from 600 to 1900 cm^−1^; (**h**) From 2800 to 3400 cm^−1^.

Figure 2 shows both the non-derivative and derivative FTIR spectra of serum from discharged and deceased patients, which were obtained on the day of outcome (i.e., the day the patient is either discharged or deceased). The average FTIR spectra of the 21 serum samples from discharged patients and 23 from deceased patients are notably similar, particularly after baseline correction and normalization (Figure 2c). Nevertheless, some spectral differences can be observed. More pronounced variations appear in both the first derivative (Figure 2d) and second derivative spectra (Figure 2e), as anticipated, since derivatives help resolve overlapping spectral bands [49].

**Figure 3:**
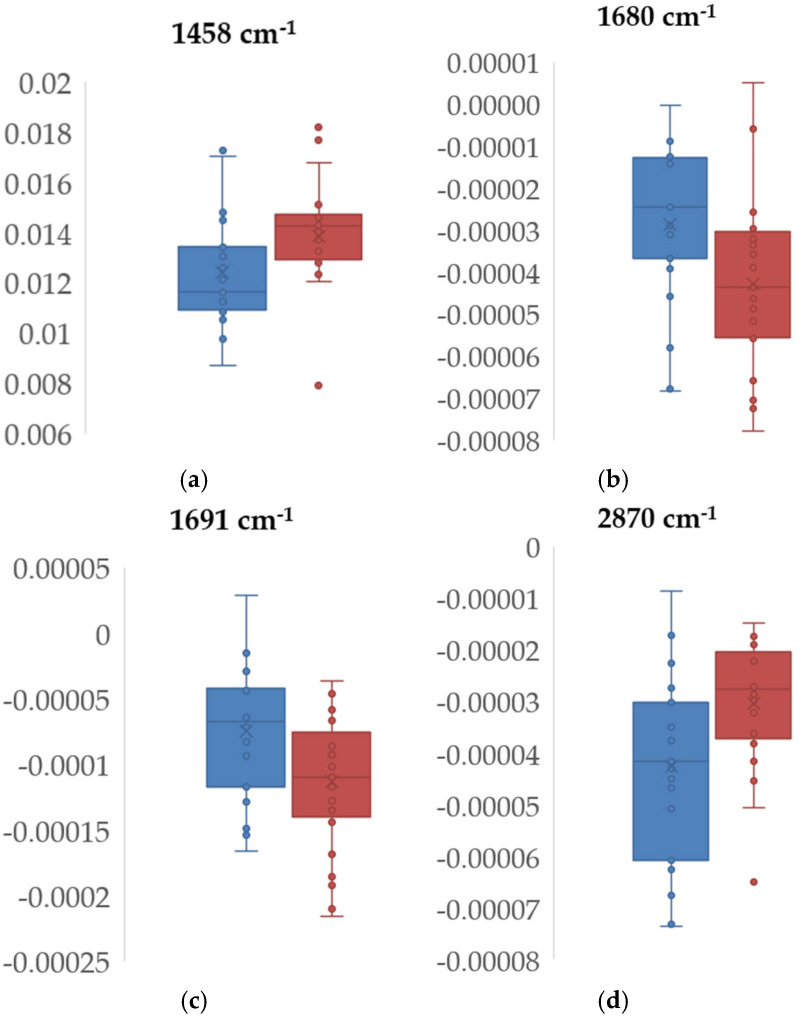
Boxplots of spectral bands of discharged (blue) and deceased (red) COVID-19 ICU patients that were statistically different between the two populations: 1458 cm^−1^ for the (**a**) normalized baseline correction and (**b**) 1680, (**c**) 1691, and (**d**) 2870 cm^−1^ for the second derivative spectra.

Based on the average normalized baseline correction, the first derivative, and the second derivative spectra of sera, a total of fourteen positive, nine positive, and twenty-four negative bands were considered, respectively (Figure 2c,d,f, Supplementary Table S1). Bands with a lower signal-to-noise ratio (SNR), which were generally observed at the extremes of the midinfrared (MIR) range (from 400 to 600 cm^−1^) and between 2200 and 2400 cm^−1^ due to atmospheric water vapor and CO_2_ bands, were excluded [50,51,52]. From all the identified bands, four showed statistically significant differences in signal intensity values between the two groups, i.e., discharged versus deceased patients (Table 2, Figure 3), one band from the normalized baseline-corrected spectra, and three bands from the second derivative spectra regions, for selected regions.

Regarding the identified bands in Table 2, the band at 1458 cm^−1^ is associated with lipids and proteins, the bands in the range of 1680–1691 cm^−1^ are commonly associated with the amide I, and the band at 2870 cm^−1^ is associated with lipids. These serum bands have been previously identified as relevant in various disease states. For example, the amide I bands (~1680–1691 cm^−1^) have been associated with sudden cardiac death [54], with some studies having linked them to amyloid diseases [55]. One study specifically identified a band at 1682 cm^−1^ while investigating immune complex recognition [56]. Furthermore, the band at 2870 cm^−1^ has been linked to changes in lipid composition, as observed in studies on skin cancer [57].

A Naïve Bayes predictive model of mortality was developed based on the three bands showing the most significant differences between the two populations in the second derivative spectra (Figure 3b–d). However, the models showed very low predictive performance, with AUC values of 0.61, 0.44, and 0.66 for the bands at 1680, 1691, and 2870 cm^−1^, respectively. Notably, combining the three bands did not improve the model’s performance, with an AUC of 0.59. While other machine learning models (kNN, SVM, Random Forest, and Decision Tree) were also applied, their results were significantly poorer than those of Naïve Bayes; hence, only the best-performing model is reported.

### 2.4. Multivariate Spectral Analysis

Following an initial univariate spectral analysis, which yielded poor results, a multivariate approach was pursued, as combining multiple biomarkers is well-established in the literature to enhance predictive accuracy in ICU settings [58,59].

Diverse unsupervised t-distributed Stochastic Neighbor Embedding (t-SNE) analyses were conducted to evaluate the impact of spectral preprocessing methods (Figure 2). For this analysis, samples taken on the day of outcome (discharge or death) were considered. t-SNE is a valuable tool for visualizing high-dimensional data, which is critical when working with FTIR spectroscopic data. It condenses variance (e.g., information within the dataset) into a lower-dimensional space while preserving the data’s intrinsic nature. This makes t-SNE particularly well-suited for revealing clusters or groupings within large and complex datasets, though its effectiveness can vary depending on the dataset.

**Figure 4:**
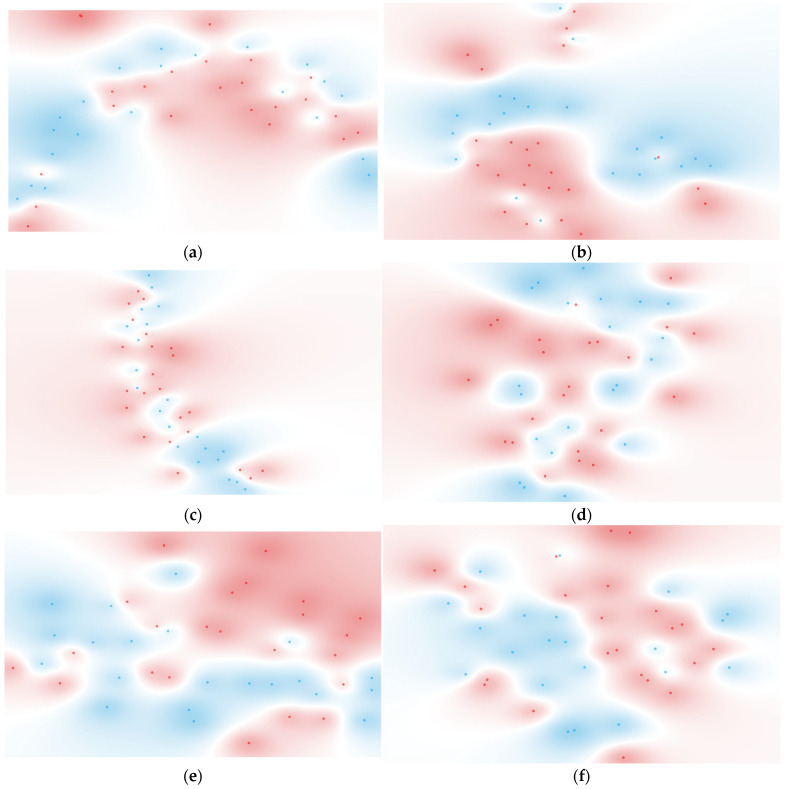
t-SNE of serum from discharged (blue) and deceased (red) patients after the following preprocessing: (**a**) None; (**b**) Baseline correction and unit vector normalization; (**c**) First derivative; (**d**) Second derivative; (**e**) Second derivative from 600 to 1900 cm^−1^; (**f**) 2800 to 3400 cm^−1^.

Some t-SNE results show a data pattern partially aligning with patient outcomes, particularly when based on spectra with baseline correction and unit-vector normalization (Figure 4b) or sub-regions of second-derivative spectra (Figure 4e,f). This suggests a degree of clustering, where the two target groups (discharged and deceased) tend to form distinguishable regions. However, complete separation between the two populations was not observed, likely reflecting the complex and overlapping nature of the metabolic signatures of critically ill ICU patients. Therefore, in the following sections, diverse supervised classification methods were applied, which were also based on various spectral preprocessing methods.

#### 2.4.1. Mortality Prediction on the Day of Outcome (ICU Discharge or Death)

The impact of various preprocessing methods on different supervised classification models was evaluated, including atmospheric compensation, baseline correction, normalized baseline correction, first derivative, and second derivative. For the second derivative spectra, both the full spectra and selected regions, as previously analyzed, were considered. Table 3 summarizes the performance of predictive models based on serum samples collected on the day of the outcome, i.e., the day patients were discharged or deceased in the ICU. For non-derived spectra, the best model was a Decision Tree using normalized baseline correction, achieving an AUC of 0.750. For derived spectra, the best results were obtained with a Decision Tree based on the first derivative spectra (AUC = 0.705).

To further improve model performance, and since all AUC values were below 0.8, feature selection based on Fast Correlation Based Filter (FCBF) was applied. FCBF is particularly suited for high-dimensional datasets, such as FTIR spectra, due to its ability to reduce entropy within models, potentially enhancing differentiation between discharged and deceased patient groups. This filtering process was applied across all preprocessing methods and supervised classification models, resulting in significant improvements (Table 4).

After applying FCBF, the best performance (determined by a combination of AUC and sensitivity, with sensitivity prioritized over specificity as it represents true positive samples, i.e., correctly identified deceased patients) for non-derived spectra was achieved with a Random Forest model built on normalized baseline-corrected spectra (AUC = 0.81). For derivative spectra, an excellent model was obtained with a Random Forest model based on second derivative spectra, achieving an AUC of 0.96, sensitivity of 0.96, and specificity of 0.76. A Naïve Bayes model based on the same second derivative spectra within the regions 600–1900 cm^−1^ and 2800–3400 cm^−1^ also excelled, demonstrating superior metrics in both AUC (0.98) and specificity (0.91) while still performing well in sensitivity (0.87).

The Naïve Bayes model was chosen for further analysis as the selected regions (600–1900 cm^−1^ and 2800–3400 cm^−1^) provided a higher SNR compared to the complete second derivative spectra. Since an excellent model was achieved for predicting mortality on the day of the outcome (i.e., the day of discharge or death), additional predictive models were developed for days increasingly distant from the outcome, i.e., from 1 to 7 days prior to the outcome, as well as for the first 48 h after ICU admission.

#### 2.4.2. Mortality Prediction from 1 to 7 Days Prior to the Day of Outcome

Diverse Naïve Bayes models were developed to predict mortality 1 to 7 days prior to the day of outcome, using the second derivative regions from 600 to 1900 cm^−1^ and 2800 to 3400 cm^−1^ (Table 5). Excellent models were achieved (AUC > 0.9) for all periods between 1 and 7 days before the outcome (discharged or deceased). These models were slightly better than the model built with samples collected on the day of the outcome. This may reflect the higher variability in serum composition on the day of the outcome due to intensive therapeutic interventions.

#### 2.4.3. Mortality Prediction at the First 48 h of ICU Admission

**Figure 5:**
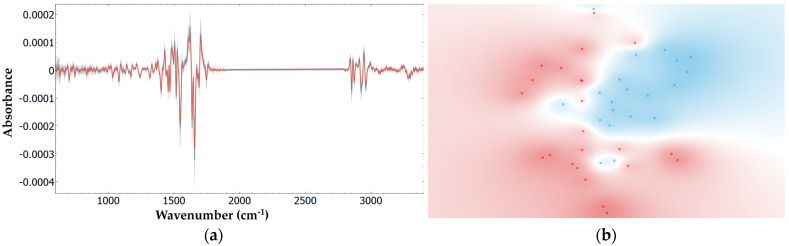
FTIR spectra and corresponding t-SNE plots of serum from 20 discharged (blue) and 23 deceased (red) patients for the following: (**a**) FTIR spectra for the second derivative between 600–1900 and 2800–3400 cm^−1^; (**b**) Its corresponding t-SNE.

Predictive models of mortality were also developed based on serum samples collected within the first 48 h of ICU admission. This approach was necessary due to the lack of admission samples for all 44 patients. Notably, one patient from the discharged group was excluded from this analysis due to the absence of samples within this timeframe. This exclusion did not negatively impact the previously reported *p*-values in Table 1, with all variables remaining above the 1% level (*p*-value > 0.01). The same preprocessing steps and Naïve Bayes model applied in earlier analyses were used here, specifically with the second derivative preprocessing applied to the region between 600–1900 cm^−1^ and 2800–3400 cm^−1^. As previously demonstrated, this approach yielded the best performance metrics, which were confirmed in this analysis, resulting in an AUC, sensitivity, and specificity of 0.980, 0.913, and 0.950, respectively. The t-SNE visualization produced from this preprocessing can be observed in Figure 5.

#### 2.4.4. Molecular Insights from Spectral Regions Identified for ICU Mortality Prediction

The development of mortality risk prediction models for critically ill patients is challenging due to the highly heterogeneous pathophysiological states of ICU patients and the marked evolution of their physiological responses over time [60,61]. For these reasons, the predictive model based on the day of the outcome (i.e., discharge or death) was developed as a starting point, as it was expected to be simpler to construct. Serum spectra from that day directly reflect metabolic disruptions caused by the most traumatic event—death.

However, for clinical utility, it is crucial to develop robust predictive models as early as possible. To address this, the model based on the day of the outcome was compared with models predicting mortality risk earlier, including at the first 48 h of ICU admission and up to seven days prior. By focusing on these key intervals, we strive to provide clinicians with relevant, time-specific indicators of patient progression toward recovery or potential mortality. It is important to note that, in this section, a final refinement step was applied to the analysis for all three timeframes. Specifically, since all preprocessing in this section pertains to the second derivative spectra, only negative peaks and minima were considered, as these are the features with direct biochemical interpretability in FTIR spectroscopy. This step was intended to enhance the robustness and clinical relevance of the findings after the optimal preprocessing and predictive models had been determined in the previous sections of this study.

The suitability and clinical utility of each timeframe can differ significantly. While the first 48 h of ICU admission provide early indicators, variability in patient conditions upon admission—including prior treatments and baseline health—can introduce confounding factors, making predictions less reliable. This variability is further compounded by the fact that the first 48 h encompass patients from both the first 24 h of ICU admission and the 24–48 h period. Unsurprisingly, these patients exhibit distinct metabolic and health states, as significant changes in care and therapeutic interventions can occur within this timeframe. This variability resulted in a decrease in well-defined negative peaks in the second derivative, as observed in Table 6. In contrast, the seven days prior to the outcome represent a critical period where metabolic and physiological changes are more stable and reflective of the patient’s progression, offering an optimal window for actionable clinical insights. Finally, the day of the outcome captures the most pronounced biochemical disruptions but has limited utility for real-time intervention due to the imminent nature of the outcome. Together, these complementary timeframes allow for a comprehensive understanding of mortality risk dynamics, with the seven-day window offering the greatest potential for early and effective clinical decision-making.

The best predictive models for mortality risk on the first 48 h after ICU admission, seven days prior to the outcome, and the day of the outcome are summarized in Table 6.

By leveraging the FTIR spectra as unique metabolic fingerprints, this study captures a comprehensive view of each patient’s biochemical state through the overall shape of the spectra rather than isolated absorbance values, which can vary due to individual, geographical, or demographic factors. Using averaged spectra for each group (discharged or deceased) as references, the models classify patients based on how closely their metabolic fingerprint aligns with these profiles, highlighting the robustness and universality of the spectral analysis.

The bands identified at each key interval provide molecular insights into the key biochemical components associated with mortality.

For instance, within the first 48 h after ICU admission, the most prominent bands identified reflect distinct metabolic components. The band around 3027 cm^−1^, associated with CH stretching, indicates alterations in lipid metabolism, often observed during systemic inflammation and energy imbalance in critically ill patients [62]. In contrast, the band around 1689 cm^−1^, linked to Amide I (C=O stretching in proteins), may signify protein conformational changes or stress responses, both of which are critical in predicting mortality outcomes in ICU patients [63].

For the seven days prior to the outcome, proteins and lipids were prominent, e.g., the lipid-protein interaction at ~1456 cm^−1^ (δ(CH_3_)) may reflect oxidative stress or alterations in lipid metabolism, responses commonly observed in deteriorating ICU patients [64,65].

Finally, on the day of the outcome, alterations in glucose (~1075 cm^−1^) and lipid metabolism were identified as key biochemical markers. In critically ill patients, increased energy demands due to inflammation and cellular stress often occur [66]. Lipid metabolism shifts (~1461 cm^−1^, due to δ(CH_3_)) suggest significant disruptions in lipid metabolism [67].

In summary, this time-specific analysis highlights the molecular changes occurring at different ICU stages, illustrating shifts in protein, lipid, and carbohydrate metabolism associated with patient outcomes. By targeting these regions, the model provides clinically meaningful insights into biochemical alterations that could aid in monitoring and intervention decisions, with future potential for validation in diverse ICU populations.

Although focused on a specific cohort, broader applications of such models are essential. Future work should expand these predictive models to other regions within Portugal, across the EU, and globally to develop datasets that reflect each population’s unique biochemical spectral signatures. Models capable of comparing information across countries [68,69] in a fast and efficient way are paramount for epidemic and pandemic control, as differences in country policies and political views should take a backseat when saving lives [70]. Given that regional biomarker variations can significantly impact predictive accuracy, creating a repository of population-specific data would greatly support pandemic preparedness [71,72]. This approach is particularly relevant as research highlights that future pandemics may spread more readily and pose heightened risks due to climate volatility and increasing urban density [73,74]. A proactive global health framework equipped with robust, localized predictive models could be invaluable in mitigating the impact of future infectious disease threats.

## 3. Materials and Methods

### 3.1. Study Population

Forty-four patients admitted to the ICU of Hospital São José in Lisbon were included in this study, which is part of the Predictive Models of COVID-19 Outcomes for Higher Risk Patients Towards a Precision Medicine (PREMO) project and approved by the hospital’s Ethics Committee. Informed consent was obtained from all patients or their immediate family members for data collection. All demographic, clinical, and laboratory data were collected from the hospital’s electronic medical records system and anonymized.

All patients included in this study (Table 1) were critically ill COVID-19 patients, confirmed via real-time polymerase chain reaction tests (RT-PCR). Patients with non-continuous, time-sampled data (i.e., missing samples on consecutive days) or incomplete datasets for the day of the outcome (discharge or death in the ICU) or up to seven days prior were excluded.

Biological samples were collected from patients admitted to the ICU between 12 November 2020 and 24 September 2021. The final dataset includes samples from three key timeframes: the first 48 h of ICU admission, 1 to 7 days prior to the day of the outcome, and the day of the outcome. This amounts to eight samples per patient, totaling 352 unique samples for the 44 patients (17 females and 27 males), along with an additional 43 samples collected during the first 48 h after ICU admission.

### 3.2. Collection of Biological Samples

Peripheral blood was collected in VACUETTE^®^ tubes without anticoagulant, using standard blood collection procedures. Serum was obtained by centrifuging the samples at 3500 rpm for 10 min (Mikro 220T, Hettich, Kirchlengern, Germany) and stored at −20 °C until FTIR spectra acquisition.

### 3.3. FTIR Spectra Acquisition and Preprocessing

Triplicates of 25 μL of serum, pre-diluted at a 1:10 ratio in water, were pipetted onto a 96-well Si plate and dehydrated for approximately 3.5 h in a vacuum desiccator (Vacuubrand, ME 2, Wertheim, Germany). Spectral data were collected using an FTIR spectrometer (Vertex 70, Bruker, Mannheim, Germany) equipped with a High-Throughput Screening eXTension (HTS-XT) accessory (Bruker, Ettlingen, Germany). Each spectrum represented 64 co-added scans at a resolution of 2 cm^−1^ and was collected in transmission mode between 400 and 4000 cm^−1^. The first well of the 96-well plate was left empty, and the corresponding spectra were acquired and used as background, following the HTS-XT manufacturer’s instructions.

The impact of the following preprocessing methods, applied after atmospheric compensation, was evaluated: baseline correction (using a rubber band baseline type for the positive peak direction with ‘subtract’ as the background action) with unit vector normalization, and first- and second-order derivatives using a Savitzky-Golay filter with a second order polynomial over a 15-point window.

### 3.4. Data and Statistical Analysis

The demographic variables in this study included age and gender, while clinical characteristics comprised body mass index (BMI), arterial hypertension, diabetes mellitus, and dyslipidemia. Types of respiratory support provided—specifically IMV, which all patients received, and ECMO—were also considered. These variables were selected for their clinical relevance in COVID-19 patients, as they are significant prognostic factors for ICU outcomes [47,48]. It was assessed whether these variables exhibited statistically significant differences between the two patient groups: discharged and deceased. Statistical significance was defined as a two-sided *p*-value of less than 0.01.

Statistical analysis of patients’ demographic and clinical characteristics was conducted (Table 1) using the Student’s *t*-test for continuous variables with a normal distribution and the Mann–Whitney U test for non-normally distributed continuous variables. For categorical data, Chi-square (χ^2^) and Fisher’s exact tests were applied, with Fisher’s test specifically used for smaller sample sizes. Continuous variables were represented as medians and interquartile ranges (25th to 75th percentiles), while categorical data were presented as absolute frequencies and percentages. All statistical analyses were performed using IBM SPSS Statistics software, version 27 (IBM Corp., New York, NY, USA).

For time series analysis, the ARIMA model was applied to predict outcomes based on observed trends in the data. The model parameters (0,1,14) represent an ARIMA configuration where ‘0’ indicates no autoregressive terms, ‘1’ denotes the order of differencing applied to make the series stationary, and ‘14’ specifies the number of lagged forecast errors in the moving average model. This configuration was chosen based on preliminary analysis to achieve the best fit for the data.

t-SNE, an unsupervised classification method [75], was conducted to visualize patient groupings (Figure 4). t-SNE is a dimensionality reduction technique that projects high-dimensional data into two or three dimensions while preserving relative distances between data points, revealing clusters or groupings in complex datasets. This method was preferred over the use of Principal Component Analysis (PCA), a satisfactory but linear technique often highly affected by the presence of outliers [76]. Unlike PCA, which maps data to new axes corresponding to linear combinations of original variables, t-SNE does not assign specific values or physical meaning to its axes. Instead, it creates a low-dimensional representation where the proximity of points reflects the local relationships and similarities in the high-dimensional data. This makes t-SNE particularly suited to capturing non-linear patterns in spectral data that may not be apparent with PCA.

Additionally, supervised classification models—k-Nearest Neighbors (kNN), Naïve Bayes, Random Forest, Support Vector Machine (SVM), and Decision Tree—were developed.

A 5-fold cross-validation was implemented, where the dataset was divided into five equal parts, with the model trained on four parts and tested on the remaining part in each iteration, ensuring each sample was tested once. This approach not only provides a robust estimate of model performance but also reduces the risk of overfitting by ensuring that no single subset of the data disproportionately influences the model’s results. Overfitting, a concern in predictive modeling, especially with smaller datasets, was further addressed through the use of feature selection via FCBF, which prioritizes features most relevant for group discrimination, and by averaging model performance across all folds of the cross-validation. While small sample sizes inherently carry the risk of overfitting, these steps were taken to mitigate this issue and ensure that the reported performance metrics accurately reflect the models’ predictive capability within this dataset.

Feature selection for spectral bands was performed using FCBF, an entropy-based algorithm that ranks spectral bands by their importance in discriminating target groups (e.g., discharged versus deceased). FCBF assigns values to each feature (i.e., wavenumber), where higher scores indicate greater importance for group discrimination [77]. This method was chosen for its proven effectiveness in biomedical applications and its ability to improve diagnostic accuracy [78]. These analyses were conducted using Orange: Data Mining Toolbox [79], version 3.36.2 (Bioinformatics Lab, University of Ljubljana, Ljubljana, Slovenia).

## 4. Conclusions

In this preliminary study, predictive models for early mortality prediction were developed for a heterogeneous ICU population. Spectral bands identified within the first 48 h after ICU admission, seven days prior to the outcome, and on the day of the outcome (i.e., discharge or death) included two, five, and eight bands, respectively. While models for the seven-day timeframe and the day of the outcome demonstrated excellent performance metrics, with AUC, sensitivity, and specificity values all above 0.87, the first 48 h timeframe presented greater variability, resulting in more modest predictive metrics. This variability stems from the heterogeneity of patient conditions upon admission, including prior or ongoing treatments as well as rapidly evolving health states during the initial critical period. The identified spectral bands represent a wide range of molecules, including DNA, proteins, and lipids, which reflect the complex and intricate mechanisms at play in critically ill ICU patients. This research underscores the importance of defining a set of biomolecules—exemplified by the molecular fingerprint of serum captured through FTIR spectroscopy—rather than relying solely on individual biomarkers. Moreover, this study highlights the advantage of utilizing standard care procedures with routinely collected blood samples, which allow for the fast and reliable acquisition of biomarkers. Additionally, spectra are acquired using a rapid, economical, and high-throughput method, making it suitable for large-scale population studies, which are critical in scenarios such as pandemics and other major societal challenges. The timeframes explored in this study—48 h after ICU admission, seven days prior to the outcome, and the day of the outcome—offer clinical bodies flexibility in monitoring the metabolic evolution of ICU patients. Depending on specific clinical goals, one or more of these timeframes could be employed to adjust predictions and interventions as patients progress. Future studies with larger cohorts from multiple hospitals and regions are necessary to validate these models and results, ensuring their clinical robustness and utility. Over time, evolving the current three timeframes into a longitudinal framework with daily monitoring could provide an almost real-time window into the patients’ metabolic state, enabling more accurate predictions while also allowing for the evaluation and adjustment of therapeutic strategies for optimizing outcomes. This study holds the potential for identifying robust biomarkers for ICU mortality prediction, which enables treatment adjustments and improved clinical management of hospital resources.

## Figures and Tables

**Table 1:** Demographic and clinical characteristics of 44 ICU patients, 23 of whom were deceased. The significance level for statistical analysis comparing the two groups was set to α = 0.01 (1%).

		Discharged Patients (*n* = 21)	Deceased Patients (*n* = 23)	*p*-Value
Age (years), median (IQR)		60 (15)	67 (15)	0.205 ●
ICU stay (days), median (IQR)		14 (12)	11 (5)	0.016 #
Body Mass Index (kg/m^2^), median (IQR)		30.85 (8.30)	27.37 (4.66)	0.036 #
Gender, *n* (%)	Female	9 (43)	8 (35)	0.811 *
	Male	12 (57)	15 (65)	
ECMO, *n* (%)	No	19 (90)	23 (100)	0.222 +
	Yes	2 (10)	0 (0)	
Arterial Hypertension, *n* (%)	No	7 (33)	3 (13)	0.155 +
	Yes	14 (67)	20 (87)	
Diabetes Mellitus, *n* (%)	No	13 (62)	13 (57)	0.955 *
	Yes	8 (38)	10 (43)	
Dyslipidemia, *n* (%)	No	13 (62)	12 (52)	0.729 *
	Yes	8 (38)	11 (48)	

Statistical tests used: ● Student’s *t*-test, # Mann–Whitney U, * Chi-square (χ^2^), + Fisher’s exact test.

**Table 2:** Average values and standard deviations for statistically significant spectral bands from the normalized baseline correction and second derivative spectra (600–1900, 2800–3400 cm^−1^) for serum samples from discharged and deceased ICU COVID-19 patients on the day of outcome, along with their molecular assignments according to [53]. The corresponding *p*-values from Welch’s *t*-test comparisons between the two populations are also presented.

		Discharged	Deceased			
Preprocessing	Bands (cm^−1^)	Average (±SD)	Average (±SD)	*p*-Value	Vibrational Mode *	Functional Group /Biocompound
Normalized baseline correction	1458	1.24 × 10^−2^ (±2.24 × 10^−3^)	1.38 × 10^−2^ (±2.40 × 10^−3^)	0.047	6as(CH_3_) 6as(CH_3_)	Lipid, protein
Second Derivative between 600–1900 and 2800–3400 cm^−1^	1680	−2.87 × 10^−5^ (±1.91 × 10^−5^)	−4.29 × 10^−5^ (±2.10 × 10^−5^)	0.023	80% ν(CO), 20% ν(CN)	Amide I peptide, protein
	1691	−7.47 × 10^−5^ (±5.22 × 10^−5^)	−1.10 × 10^−4^ (±5.37 × 10^−5^)	0.020	80% ν(CO), 20% ν(CN)	Amide I peptide, protein
	2870	−4.26 × 10^−5^ (±1.89 × 10^−5^)	−3.05 × 10^−5^ (±1.27 × 10^−5^)	0.019	νs(CH_3_)	Lipids

* ν, stretching; δ, bending; as, antisymmetric; s, symmetric.

**Table 3:** Performance of 5-fold cross-validation for discriminating discharged and deceased ICU COVID-19 patients on the day of the outcome, using various spectral preprocessing methods and machine learning models. The analysis includes the complete spectral range (400–4000 cm^−1^) unless otherwise specified, with performance metrics reported only for the deceased group.

Spectra Preprocessing	Model	AUC	Sensitivity	Specificity
Atmospheric Compensation	kNN	0.557	0.565	0.524
	Naïve Bayes	0.490	0.348	0.429
	Random Forest	0.581	0.696	0.476
	SVM	0.390	0.696	0.429
	Decision Tree	0.495	0.565	0.286
Normalized baseline correction	kNN	0.723	0.696	0.714
	Naïve Bayes	0.670	0.652	0.524
	Random Forest	0.714	0.870	0.571
	SVM	0.610	0.652	0.714
	Decision Tree	0.750	0.783	0.714
First Derivative	kNN	0.642	0.435	0.714
	Naïve Bayes	0.512	0.435	0.429
	Random Forest	0.637	0.609	0.429
	SVM	0.380	0.609	0.429
	Decision Tree	0.705	0.696	0.714
Second Derivative	kNN	0.628	0.522	0.667
	Naïve Bayes	0.640	0.435	0.667
	Random Forest	0.455	0.565	0.476
	SVM	0.410	0.783	0.524
	Decision Tree	0.660	0.696	0.571
Second Derivative between 600–1900 and 2800–3400 cm^−1^	kNN	0.617	0.478	0.524
	Naïve Bayes	0.565	0.478	0.619
	Random Forest	0.681	0.696	0.524
	SVM	0.400	0.783	0.524
	Decision Tree	0.490	0.565	0.429

Abbreviations: kNN: k-Nearest Neighbors; SVM: Support Vector Machine.

**Table 5:** Performance of 5-fold cross-validation for Naïve Bayes prediction models for discriminating discharged and deceased ICU COVID-19 patients, based on serum spectra acquired 1 to 7 days prior to the outcome. The analysis was based on the second derivative spectra between 600–1900 and 2800–3400 cm^−1^ after feature selection by FCBF.

Days Prior to Outcome	AUC	Sensitivity	Specificity
1	1.000	1.000	0.952
2	0.978	0.957	0.905
3	0.957	0.957	0.857
4	0.980	0.913	0.952
5	0.980	0.913	1.000
6	1.000	0.957	1.000
7	1.000	1.000	0.952

**Table 6:** Performance of 5-fold cross-validation for Naïve Bayes prediction models discriminating discharged and deceased ICU COVID-19 patients, evaluated 48 h after ICU admission, 7 days prior to the outcome, and the day of outcome (death or discharge). Models were obtained after feature selection by FCBF and based on second derivative serum spectra.

Timeframe	Bands (cm^−1^)	AUC	Sensitivity	Specificity
48 h after ICU admission	3027; 1689	0.791	0.783	0.650
7 days from outcome	1456; 1676; 3314; 1874; 1285	0.965	0.957	0.905
Day of outcome	1075; 658; 1461; 3374; 3384; 1568; 1496; 920	0.980	0.870	0.905

**Table 4:** Performance of 5-fold cross-validation for discriminating discharged and deceased ICU COVID-19 patients on the day of the outcome. It is based on various spectral preprocessing methods and machine learning models after applying FCBF for feature selection.

Spectra Preprocessing	Bands (cm^−1^)	Model	AUC	Sensitivity	Specificity
Atmospheric Compensation	1544; 554	kNN	0.660	0.435	0.714
		Naïve Bayes	0.847	0.783	0.619
		Random Forest	0.772	0.739	0.667
		SVM	0.260	0.739	0.429
		Decision Tree	0.679	0.652	0.619
Normalized baseline correction	1610; 2570; 3398; 3990; 2206	kNN	0.820	0.696	0.714
		Naïve Bayes	0.843	0.696	0.714
		Random Forest	0.810	0.739	0.619
		SVM	0.780	0.739	0.667
		Decision Tree	0.730	0.652	0.714
First Derivative	995; 940; 1012; 1454; 3741; 2931; 494	kNN	0.515	0.565	0.429
		Naïve Bayes	0.935	0.783	0.952
		Random Forest	0.950	0.826	0.810
		SVM	0.828	0.826	0.714
		Decision Tree	0.779	0.826	0.619
Second Derivative	3511; 2232; 1075; 2617; 658; 3374; 2628; 2275; 574; 2749; 1568	kNN	0.785	0.739	0.810
		Naïve Bayes	0.980	0.870	0.952
		Random Forest	0.960	0.957	0.762
		SVM	0.915	0.739	0.762
		Decision Tree	0.749	0.783	0.714
Second Derivative between 600–1900 and 2800–3400 cm^−1^	1075; 658; 1461; 3374; 3384; 1568; 1496; 920	kNN	0.905	0.826	0.762
		Naïve Bayes	0.980	0.870	0.905
		Random Forest	0.960	0.913	0.857
		SVM	0.948	0.826	0.857
		Decision Tree	0.654	0.652	0.667

Abbreviations: kNN: k-Nearest Neighbors (kNN); SVM: Support Vector Machine.

## Data Availability

The data presented in this study are not publicly available due to privacy or ethical restrictions and due to the ongoing nature of the study.

## Supplementary Materials

The supporting information can be downloaded at https://www.mdpi.com/article/10.3390/ijms252413609/s1.
