# Supplementary material for: Early Mortality Prediction in Intensive Care Unit Patients Based on Serum Metabolomic Fingerprint
Source: Int J Mol Sci. 2024 Dec 19;25(24):13609. doi: 10.3390/ijms252413609 (PMC11677344; doi:10.3390/ijms252413609)
Supplement: Supplementary file 1 [file ijms-25-13609-s001.zip › ijms-3354523-supplementary.pdf]

International Journal of
Molecular Sciences

MDPI

Supplementary Table S1. Identified spectral bands for the three preprocessing methods considered for the univariate analysis, with the respective *p*-values from Welch's *t*-test. In a gray background, the statistically significant bands can be observed per preprocessing, when applicable.

**Normalized baseline correction**		**First Derivative**		**Second Derivative**	
Region: 400-4000 cm^{-1}		400-4000 cm^{-1}		600-1900, 2800-3400 cm^{-1}	
**Bands (cm^{-1})**	***p*-value**	**Bands (cm^{-1})**	***p*-value**	**Bands (cm^{-1})**	***p*-value**
702	0.127	1026	0.400	618	0.225
835	0.243	1226	0.904	702	0.493
1079	0.085	1387	0.271	747	0.677
1244	0.643	1512	0.490	836	0.108
1316	0.528	1533	0.269	1080	0.115
1402	0.294	1631	0.426	1173	0.159
1458	0.047	2848	0.594	1317	0.633
1548	0.333	2917	0.649	1402	0.511
1655	0.527	3260	0.611	1440	0.190
2854	0.549			1456	0.808
2871	0.367			1469	0.717
2928	0.803			1517	0.702
2960	0.320			1547	0.164
3300	0.469			1639	0.908
				1658	0.164
				1680	0.023
				1691	0.020
				1748	0.240
				2853	0.520
				2870	0.019
				2925	0.560
				2960	0.985
				3300	0.149
				3345	0.308
